# 磺酸功能化聚合离子液体基磁性吸附剂的制备及对敌草快的萃取

**DOI:** 10.3724/SP.J.1123.2022.01027

**Published:** 2022-10-08

**Authors:** Bingzhi GUO, Zhen YANG, Yaming SUN, Lijun HE

**Affiliations:** 1.河南工业大学化学化工学院, 河南 郑州 450001; 1. School of Chemistry and Chemical Engineering, Henan University of Technology, Zhengzhou 450001, China; 2.郑州市复杂体系精准分离分析重点实验室, 河南 郑州 450001; 2. Key Laboratory of Accurate Separation and Analysis for Complex Matrix of Zhengzhou City, Zhengzhou 450001, China

**Keywords:** 磺酸功能化聚合离子液体, 吸附剂, 磁性固相萃取, 高效液相色谱, 敌草快, sulfonic acid-functionalized polymeric ionic liquid, adsorbent, magnetic solid-phase extraction, high performance liquid chromatography (HPLC), diquat

## Abstract

有效萃取复杂食品样品中的极性污染物是实现其准确测定的瓶颈,也是食品安全分析的难点。针对污染物的结构特点,设计和发展能与之产生特定作用的新型材料是高效萃取的关键。敌草快是一种广谱性除草剂,为碱性的阳离子有机化合物。该文以1-乙烯基咪唑和1,3-丙磺酸内酯为原料合成了磺酸功能化的离子液体,通过自由基聚合反应,将其固载至磁性纳米颗粒表面,得到磺酸功能化的聚(1-乙烯基-3-丙基磺酸基咪唑氯盐)修饰的磁性纳米颗粒(Poly([VPImi-SO_3_H][Cl])-MP)。采用红外光谱、扫描电镜、振动样品磁强计和热重分析等对其结构、形貌和磁性进行了表征。将其作为磁性固相萃取的吸附剂,萃取青菜中的敌草快。磺酸基的功能化使Poly([VPImi-SO_3_H][Cl])-MP的表面在一定pH下带有丰富的负电荷,与敌草快之间产生强的静电吸引作用,可实现对敌草快的有效萃取。对影响萃取效率的各种参数如溶液pH、吸附剂质量、吸附时间、解吸剂种类和体积等进行了优化。在优化条件下,结合磁性固相萃取和高效液相色谱技术,对方法的性能及适用性进行了考察。敌草快在0.2~20 μg/g内具有良好的线性(*r*=0.9981),检出限(*S/N*=3)和定量限(*S/N*=10)分别为0.09 μg/g和0.2 μg/g; 3个水平(0.5、1.0和2.5 μg/g)下的加标回收率为82.7%~97.5%,相对标准偏差为2.8%~5.0%(*n*=3)。结果表明,磺酸功能化的Poly([VPImi-SO_3_H][Cl])-MP能快速、有效地萃取敌草快,建立的方法能用于青菜中敌草快的准确测定。

## Abstract (en)

In terms of the structure of polar pollutants, the design and development of new materials that can interact with target analytes is vital for effective extraction. Diquat is a broad-spectrum herbicide with strong toxicity. It is sprayed during the growing process of vegetables because of its high efficiency and low cost. Furthermore, diquat is a polar and basic cationic organic compound. Therefore, it is necessary to develop adsorbents that can form strong interaction with diquat to extract it from complex vegetable samples. In this study, sulfonic acid-functionalized ionic liquid, 1-vinyl-3-propyl(3'-sulfonate) imidazolium chloride ([VPImi-SO_3_H][Cl]), was synthesized through the reaction of 1-vinylimidazole and 1,3-propanesultone and characterized using nuclear magnetic-resonance spectroscopy. The resulting [VPImi-SO_3_H][Cl] was then immobilized on the surface of vinyl-modified magnetic silica to obtain poly-(1-vinyl-3-propyl(3'-sulfonate) imidazolium chloride)-modified magnetic nanoparticles (Poly([VPImi-SO_3_H][Cl])-MP) *via* free radical polymerization between vinyl groups. The structure, morphology and magnetism of the obtained Poly([VPImi-SO_3_H][Cl])-MP were characterized using Fourier transform infrared spectroscopy, scanning electron microscope, vibrating sample magnetometer, and thermogravimetric analysis. The characterization results indicated that the Poly([VPImi-SO_3_H][Cl])-MP was synthesized with strong magnetic properties.

Poly([VPImi-SO_3_H][Cl])-MP as magnetic adsorbent exhibited a different pH response to anion and cation. With the increase in solution pH, the adsorption efficiency of Poly([VPImi-SO_3_H][Cl])-MP for anionic dye amaranth decreased; whereas, the adsorption efficiency for cationic dye, methylene blue, increased. Under a high pH condition, sulfonic acid groups on the surface of Poly([VPImi-SO_3_H][Cl])-MP existed in the form of -SO3-. Thus, the adsorption efficiency of Poly([VPImi-SO_3_H][Cl])-MP for methylene blue could reach 95.2% due to the strong electrostatic attraction between adsorbent and positively charged methylene blue; whereas, negatively charged amaranth could hardly be adsorbed due to the electrostatic repulsion between adsorbent and amaranth. The above results showed sulfonic acid modification endowed Poly([VPImi-SO_3_H][Cl])-MP with abundant negative charges at certain pH conditions. Thus, the extraction can be achieved by the strong electrostatic attraction between negatively charged Poly([VPImi-SO_3_H][Cl])-MP and positively charged diquat. Using green vegetables as the sample matrix and magnetic solid-phase extraction as extraction model, the extraction performance of Poly([VPImi-SO_3_H][Cl])-MP for diquat was studied. Some extraction parameters affecting the extraction efficiency, such as solution pH, adsorbent amount, adsorption time, desorption solvent and its volume, and desorption time were optimized using the single factor experiment method. Under the optimized extraction conditions (30 mg of adsorbent, 15 min of adsorption time, 40 μL of ammonia, 600 μL of acetonitrile-formic acid (9∶1, v/v) as desorption solvent, 1 min of desorption time), the performance, and applicability of the proposed method were investigated by combining magnetic solid-phase extraction, high performance liquid chromatography, and UV-visible detector. Good linearity was observed at contents ranging from 0.2 to 20 μg/g with a correlation coefficient (*r*) of 0.9981. The limit of detection and limit of quantification based on the signal-to-noise ratio of 3∶1 and 10∶1 were 0.09 and 0.2 μg/g, respectively. The spiked recoveries at three levels of 0.5, 1.0, and 2.5 μg/g were obtained in the range of 82.7%-97.5% with the relative standard deviations of 2.8%-5.0% (*n*=3). The results showed that the sulfonic acid-functionalized Poly([VPImi-SO_3_H][Cl])-MP could be employed as a magnetic adsorbent for the quick and effective extraction of diquat, and the proposed method could be used for the determination of diquat from green vegetable samples. It could be anticipated that Poly([VPImi-SO_3_H][Cl])-MP could be used as an adsorbent for extracting other cationic polar pollutants by adjusting the solution pH. This work provides a new way to construct adsorbents for extracting polar pollutants.

由于基质复杂、目标分析物含量低,食品样品中微量/痕量极性污染物的准确分析一直是食品安全分析的难点和热点内容,而发展简单、高效的样品前处理技术,有效地从食品基质中萃取目标分析物是准确分析的瓶颈^[1,2]^。敌草快是一种广谱性除草剂,用途很广,常在蔬菜的生长过程中喷洒使用,但毒性强,大量使用会对人类健康造成威胁,且目前并没有针对敌草快中毒的特效药^[3]^。因此,准确检测蔬菜中敌草快的残留具有十分重要的意义。为解决样品基质复杂和目标物浓度低等问题,在检测前需使用样品前处理技术^[4,5]^。磁性固相萃取(magnetic solid-phase extraction, MSPE)作为一种新型的样品前处理技术,由于操作过程简单、便捷,萃取速度快,在食品、环境和生命科学领域受到广泛关注^[6⇓-8]^。根据目标分析物的结构特点,设计和发展能与其产生特定相互作用力的吸附介质是MSPE有效萃取目标分析物的关键。

聚合离子液体(polymeric ionic liquids, PIL)是带重复离子液体单元的高分子聚电解质,具有离子液体单体的结构可设计性以及聚合物中的重复单元能为目标物提供丰富的作用位点等特性^[9,10]^,作为磁性吸附剂已被成功用于复杂样品中农药、抗生素和真菌毒素等的有效萃取^[11⇓-13]^。作为萃取介质,PIL的最大特点是可通过调整PIL结构中的阴/阳离子,使其与目标物之间按需产生静电、疏水和*π-π*作用等,从而实现对样品中目标物的有效萃取和分离。敌草快为季铵盐类化合物,极性强,作为碱性的阳离子有机化合物,在一定的pH范围下,能电离成为高度溶剂化、质子化的阳离子,故通常使用极性溶剂将其从食品样品中提取出来,而提供疏水作用的亲脂性吸附剂对敌草快吸附能力低^[14]^,难以实现对极性提取液中敌草快的有效萃取。利用敌草快在水溶液中的阳离子特性,Kumari等^[15]^制备了4-磺酸基杯[8]芳烃改性的磁性材料,其对敌草快有高的吸附性能,能作为吸附剂用于水中敌草快的去除。Tan等^[16]^将磺酸功能化的多级孔共价有机框架材料作为表面辅助激光解吸/电离飞行时间质谱的基质和吸附剂,用于敌草快的测定,此共价有机框架材料可通过静电吸引作用高效富集敌草快。由此推测,对PIL进行磺酸功能化,通过磺酸功能基团赋予吸附剂的丰富负电荷与带正电荷的敌草快之间的强相互作用,有望实现对食品中敌草快的有效萃取。

在本实验中,拟合成磺酸功能化的离子液体单体,进一步制备磺酸功能化PIL修饰的磁性吸附剂,结合MSPE和高效液相色谱(HPLC),萃取和测定青菜中的敌草快。对影响MSPE过程的各种参数如溶液pH、吸附剂用量、吸附时间、解吸剂的种类和体积等进行了优化,并对方法性能和适用性进行了考察。

## 1 实验部分

### 1.1 仪器、试剂与材料

LC-10AT高效液相色谱仪(日本岛津公司),配SPD-10紫外检测器和7725i手动进样阀(美国罗丹尼公司), WQF-510傅里叶变换红外光谱仪与UV-2450紫外分光光度计(日本岛津公司), 7404振动样品磁强计(VSM,美国Lake Shore公司), SU8020场发射扫描电子显微镜(日本日立公司), STA 449 F3同步热分析仪(德国耐驰公司)。

1-乙烯基咪唑、1,3-丙磺酸内酯、1-乙烯基三乙氧基硅烷、正硅酸乙酯和偶氮二异丁腈均购自上海阿拉丁科技股份有限公司,甲醇、乙腈、乙醇和浓盐酸购自山东蜀王实业有限公司,FeCl_3_·6H_2_O和FeSO_4_·7H_2_O购自天津市光复精细化工研究所,氢氧化钠购自西陇化工股份有限公司,柠檬酸钠购自天津市风船化学试剂科技有限公司。敌草快(>99%)、亚甲基蓝(>99%)和苋菜红(>99%)购自上海麦克林生化科技有限公司。

色谱条件:Agilent C18色谱柱(150 mm×4.6 mm, 5 μm);柱温25 ℃;流动相为水-乙腈-甲醇混合液(95∶3∶2, v/v/v,磷酸调pH为2.8);流速1 mL/min;检测波长为256 nm。

### 1.2 磺酸功能化PIL基磁性吸附剂的制备

#### 1.2.1 乙烯基修饰的磁性纳米颗粒的制备

按照实验室以前的工作^[17]^,通过共沉淀法合成Fe_3_O_4_磁性纳米颗粒,用正硅酸乙酯对Fe_3_O_4_进行修饰得到Fe_3_O_4_@SiO_2_,用乙烯基三乙氧基硅烷对其进一步修饰得到乙烯基修饰的磁性纳米颗粒。

依次将2.8 g FeSO_4_·7H_2_O、5.2 g FeCl_3_·6H_2_O和0.85 mL浓盐酸溶于去离子水中,配制成25 mL的铁标准溶液。量取250 mL NaOH溶液(0.5 mol/L)于500 mL三口瓶中,通入氮气,机械搅拌下升温至80 ℃,逐滴滴加铁标准溶液后继续搅拌1 h,冷却至室温,逐滴滴加柠檬酸钠溶液(0.3 mol/L)100 mL,机械搅拌1 h。磁性分离后,产物用水洗涤至中性,冷冻干燥24 h,得到Fe_3_O_4_磁性纳米颗粒。

在0.29 g Fe_3_O_4_磁性纳米颗粒中加入80 mL乙醇,超声分散10 min,机械搅拌下依次加入12 mL去离子水、6 mL氨水和0.9 mL正硅酸乙酯,反应5 h。用去离子水将产物洗至中性,在0.1 mol/L HCl溶液中浸泡12 h进行酸化,将产物洗涤至中性,冷冻干燥12 h,得到Fe_3_O_4_@SiO_2_。

依次加入乙烯基三乙氧基硅烷(2 mL)、重蒸甲苯(20 mL)和三乙胺(0.3 g)于Fe_3_O_4_@SiO_2_ (3 g)中,氮气保护下升温至115 ℃,回流24 h,冷却至室温,分别使用甲苯、甲醇洗涤3次,真空干燥24 h,得到乙烯基三乙氧基硅烷修饰的磁性颗粒(vinyl triethoxysilane-modified Fe_3_O_4_@SiO_2_, Fe_3_O_4_@SiO_2_@VTES)。

#### 1.2.2 磺酸基功能化离子液体单体的合成

将0.02 mol的1-乙烯基咪唑(1.88 g)和1,3-丙烷磺酸内酯(2.44 g)加入30 mL丙酮中,40 ℃下磁力搅拌17 h。冷却至室温,抽滤后依次用丙酮和乙醇洗涤3次,60 ℃真空干燥12 h,用10 mL浓盐酸酸化5 h, 50 ℃真空干燥12 h,得到1-乙烯基-3-丙基(3'-磺酸基)咪唑氯盐(1-vinyl-3-propyl(3'-sulfonate)imidazolium chloride, [VPImi-SO_3_H][Cl])。

以氘代二甲基亚砜为溶剂,采用^1^H NMR对[VPImi-SO_3_H][Cl]进行表征,化学位移为:9.53 (H, imidazole-H), 8.21(H, imidazole-H), 7.94 (H, imidazole-H), 7.31~7.26 (H, CH=CH_2_), 5.99~5.94 (H, CH=CH_2_), 5.43~5.38 (H, CH=CH_2_), 4.37~4.30 (2H, CH_2_), 2.50~2.45 (2H, CH_2_), 2.18~2.09 (2H, CH_2_)。

#### 1.2.3 磺酸功能化PIL基磁性吸附剂的制备

通过蒸馏沉淀聚合的方式将磺酸基功能化离子液体原位共价聚合在磁性颗粒上,具体过程为:将Fe_3_O_4_@SiO_2_@VTES(0.3 g)和[VPImi-SO_3_H][Cl](0.1 g)置于20 mL甲醇中,加入10 mg偶氮二异丁腈作为引发剂,超声15 min,通入氮气,室温下机械搅拌1 h,升温至80 ℃持续搅拌至甲醇被全部蒸出,依次用甲醇、水和甲醇洗涤产物,冷冻干燥24 h,得到聚(1-乙烯基-3-丙基(3'-磺酸基)咪唑氯盐)修饰的磁性颗粒(poly-(1-vinyl-3-propyl (3'-sulfonate) imidazolium chloride)-modified magnetic particles, Poly([VPImi-SO_3_H][Cl])-MP)。整个制备过程见图1。

**图1:**
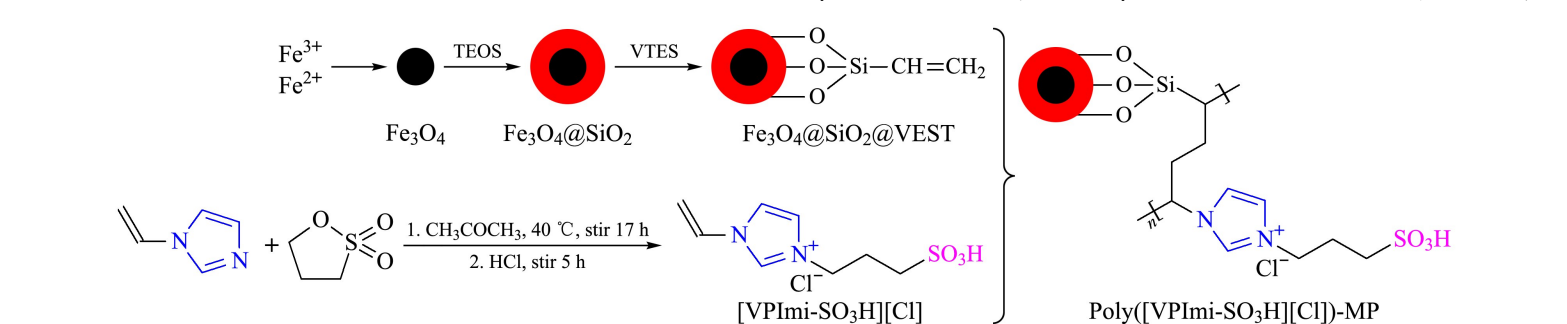
Poly([VPImi-SO_3_H][Cl])-MP的合成示意图

### 1.3 标准溶液配制、样品制备和MSPE过程

准确称取一定质量的敌草快标准品,用0.1 mol/L的HCl溶液配制成100 mg/L的标准储备溶液,放入冰箱冷藏备用。准确移取一定体积的标准储备溶液,分别用去离子水稀释、定容,得到1、2、5、10、20、50和100 mg/L的标准溶液,待用。

青菜样品购自当地超市,洗去泥土后用榨汁机将其打碎,放入冰箱冷藏备用。以没有检测出敌草快的青菜样品作为空白样品,在5.0 g空白样品中分别加入1 mL不同浓度的标准溶液,得到加标水平为0.2、0.4、1.0、2.0、4.0、10和20 μg/g的样品,用于方法性能考察。

将加标水平为2.5 μg/g的青菜样品用于萃取过程研究,称取5.0 g加标样品,加入10 mL水振荡15 min,离心3 min;转移上层提取液。提取液中加入40 μL氨水和30 mg吸附剂,振荡15 min,用磁铁分离,弃去上清液;加入600 μL乙腈-甲酸(9∶1, v/v),超声解吸1 min,用磁铁分离;上清液经0.22 μm膜过滤后,取10 μL进HPLC仪分析(见图2)。

**图2:**
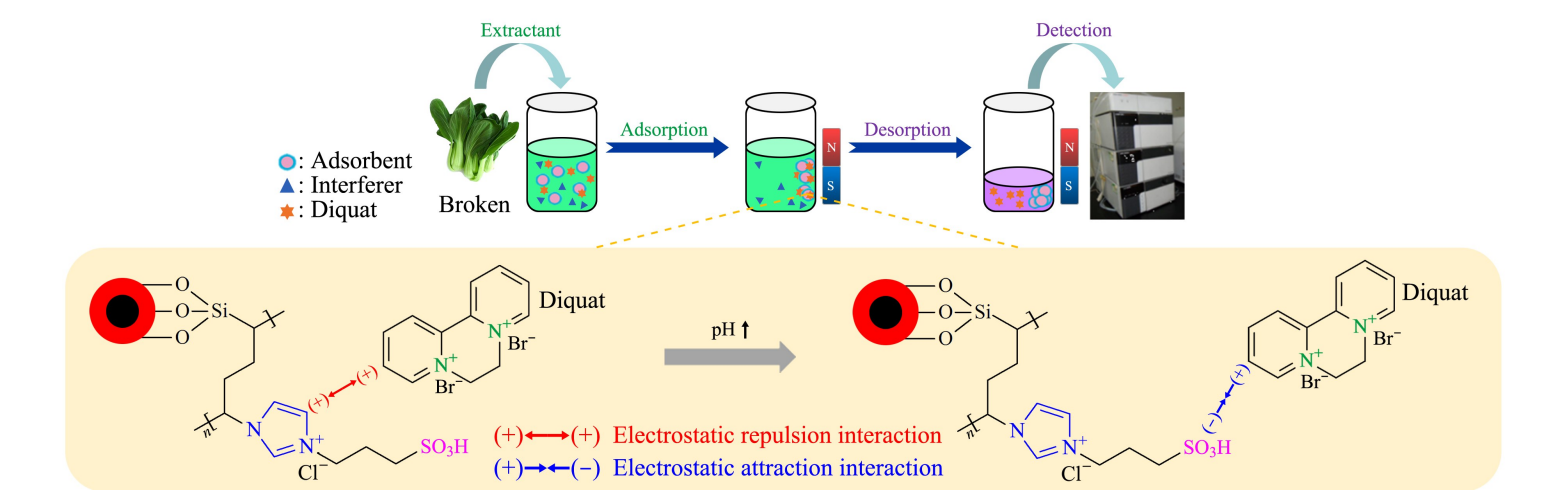
磁性固相萃取过程及其萃取机制示意图

## 2 结果与讨论

### 2.1 表征

图3a为Fe_3_O_4_@SiO_2_、Fe_3_O_4_@SiO_2_@VTES和Poly([VPImi-SO_3_H][Cl])-MP的红外光谱表征图。对Fe_3_O_4_@SiO_2_而言,在559 cm^-1^有Fe-O的特征峰,在1091 cm^-1^处有较强的Si-O特征峰,在3300 cm^-1^附近的吸收峰由硅胶表面的-OH伸缩振动引起。Fe_3_O_4_@SiO_2_@VTES在1091 cm^-1^与3300 cm^-1^附近的吸收峰强度弱于Fe_3_O_4_@SiO_2_,表明磁性颗粒表面的-OH参与了与硅烷化试剂的反应;同时,在2981 cm^-1^附近出现新的吸收峰,这是碳碳双键上C-H的伸缩振动引起的,这些结果表明乙烯基三乙氧基硅烷被成功地修饰在Fe_3_O_4_@SiO_2_上。Poly([VPImi-SO_3_H][Cl])-MP在1550 cm^-1^和1456 cm^-1^附近出现的新吸收峰归属于咪唑环的骨架振动^[17]^,这表明[VPImi-SO_3_H][Cl]已经成功键合至磁性颗粒表面。需指出的是,磺酸基在1190 cm^-1^与1068 cm^-1^左右处的特征峰被较强的Si-O特征峰覆盖。

**图3:**
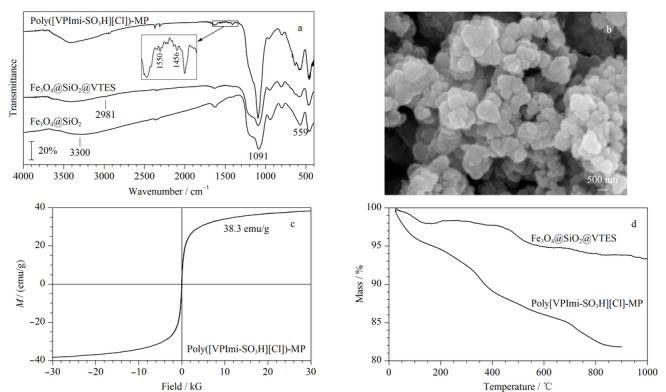
Poly([VPImi-SO_3_H][Cl])-MP的(a)红外光谱图、(b)扫描电镜图、(c)VSM曲线和(d)热重曲线图

Poly([VPImi-SO_3_H][Cl])-MP的形貌和颗粒尺寸采用扫描电镜进行表征,如图3b所示,大部分磁性颗粒尺寸在50 nm左右,形貌较规则;能谱分析显示除了C、N和O外,还存在S,这证实磺酸功能化的离子液体被成功修饰至磁性颗粒表面。图3c是Poly([VPImi-SO_3_H][Cl])-MP的磁强度表征,其VSM曲线经过原点,饱和磁强度为38.3 emu/g,说明该材料具有顺磁性,具有相当大的磁性,能方便地通过外界磁场将其与溶液分离,可以用作MSPE的吸附剂。图3d是Fe_3_O_4_@SiO_2_@VTES和Poly([VPImi-SO_3_H][Cl])-MP的热重曲线图,在25~200 ℃之间的质量损失是由水的蒸发引起的,在200~800 ℃间Fe_3_O_4_@SiO_2_@VTES质量下降是由包裹在颗粒外层VTES的质量损失所致;Poly([VPImi-SO_3_H][Cl])-MP在200~800 ℃间的质量损失要远大于Fe_3_O_4_@SiO_2_@VTES,这可能是由修饰在最外层的PIL损失导致的,这也表明PIL已成功修饰在磁性颗粒上。

### 2.2 Poly([VPImi-SO_3_H][Cl])-MP在不同pH下的吸附性能

为了评价Poly([VPImi-SO_3_H][Cl])-MP能否有效吸附带正电荷的敌草快以及可能的吸附机理,实验首先选取了苋菜红与亚甲基蓝2种染料分别作为阴离子和阳离子型分析物,直观地考察了该吸附剂在不同pH条件下的吸附性能。

从图4可以看出,Poly([VPImi-SO_3_H][Cl])-MP对阴离子染料苋菜红的吸附效率随着pH的升高而降低(曲线a),而对阳离子染料亚甲基蓝的吸附效率却随着样品溶液pH的增加而增加(曲线b),这表明Poly([VPImi-SO_3_H][Cl])-MP作为吸附剂时,其吸附性能表现出pH响应性,且对阴/阳离子的响应性不同。Poly([VPImi-SO_3_H][Cl])-MP的咪唑环带正电荷,其侧链末端的磺酸基团随溶液pH变化将以不同形式存在。当溶液pH较大时,磺酸基团主要以- ${\mathrm{SO}}_{3}^{-}$ 形式存在,与阳离子型染料亚甲基蓝之间存在强的静电吸引作用,吸附效率高达95.2%;而对阴离子型染料产生静电排斥作用,此排斥作用屏蔽了咪唑环对阴离子型染料苋菜红的静电吸引作用,使苋菜红几乎不能被吸附。当溶液pH较低时,显电中性的-SO_3_H增多,对阳离子型染料亚甲基蓝而言,静电吸引作用减弱,则吸附效率显著降低;另一方面,显电中性的-SO_3_H减弱了静电排斥作用导致的对咪唑环的屏蔽作用,使得阴离子型染料苋菜红能与咪唑环产生静电引力作用,吸附效率稍有提高。由此可知,Poly([VPImi-SO_3_H][Cl])-MP结构中磺酸功能基团的存在,不仅使该吸附剂的吸附性能表现出pH响应性,而且有望通过pH调控实现能以阳离子形式存在的敌草快的有效萃取。

**图4:**
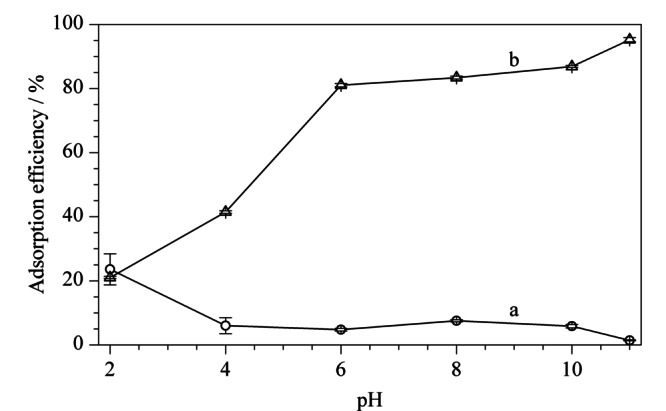
不同pH条件下Poly([VPImi-SO_3_H][Cl])-MP对(a)苋菜红和(b)亚甲基蓝的吸附效果

### 2.3 Poly([VPImi-SO_3_H][Cl])-MP萃取敌草快

#### 2.3.1 各种吸附参数对萃取效率的影响

由2.2节可知,Poly([VPImi-SO_3_H][Cl])-MP表面电荷会随着溶液pH的变化而发生改变,从而对带电荷目标物的萃取效率产生大的影响。实验通过在萃取过程中加入不同体积氨水考察溶液pH值对敌草快萃取性能的影响,结果如图5a所示。由图可知,当加入的氨水体积从5 μL增大到40 μL时,敌草快的萃取效率逐渐增加;进一步增加氨水体积,萃取效率减小。随着氨水体积的增加,溶液pH增大,吸附剂表面以-S $O_{3}^{-}$ 形式存在的磺酸基团增多,与敌草快之间的静电吸引作用增强,萃取效率增加。但由于敌草快在酸性和中性条件下才稳定,当pH值较大时,可能会加速敌草快的水解,使萃取效率降低。实验选择氨水体积为40 μL。

**图5:**
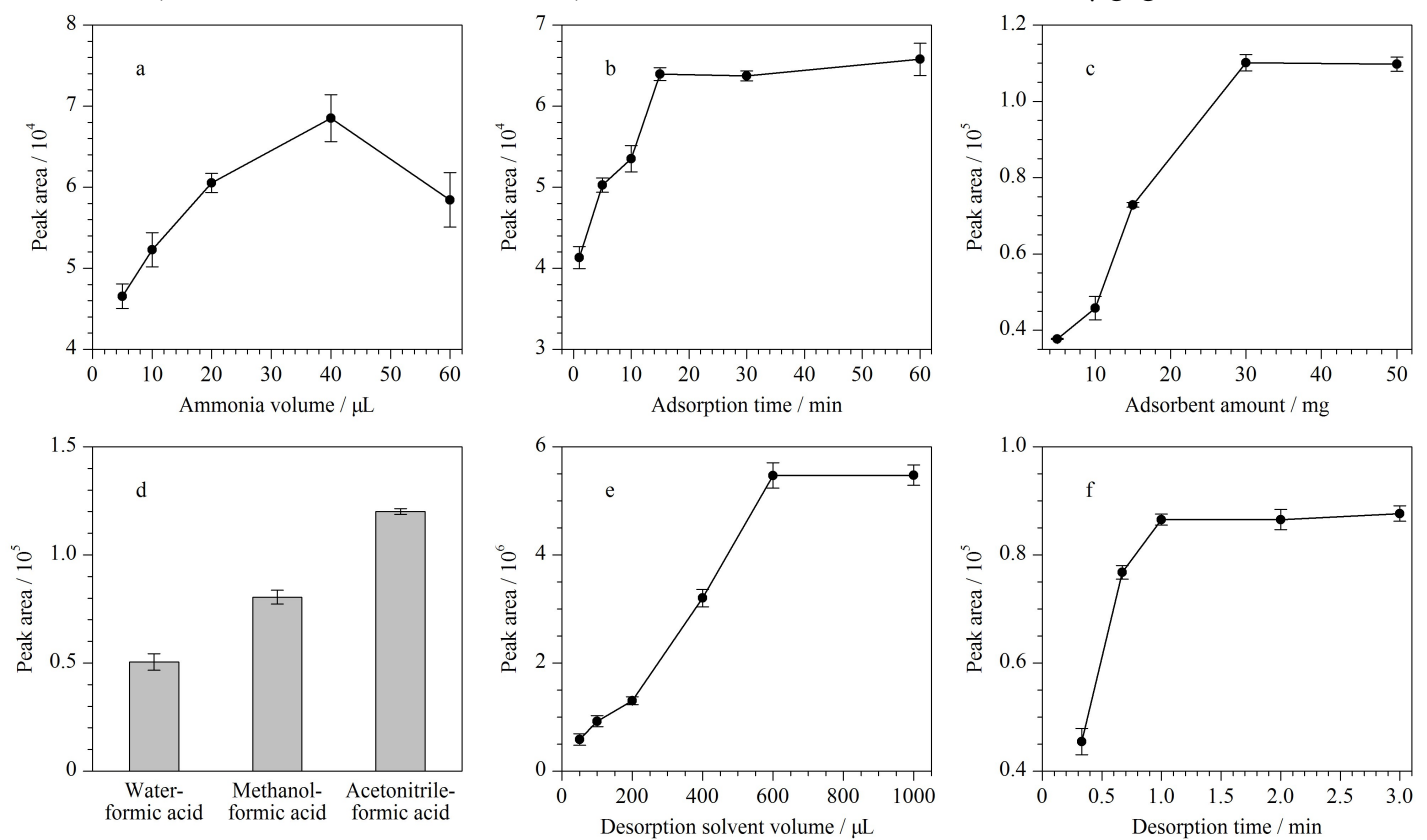
(a)氨水体积、(b)吸附时间、(c)吸附剂用量、(d)解吸剂种类、(e)解吸剂体积和(f)解吸时间对萃取效率的影响(*n*=3)

吸附时间是MSPE中影响萃取效率的一个重要因素,同时也是评价吸附剂性能的一个重要指标。实验考察了吸附时间(1~60 min)对萃取效率的影响,结果如图5b所示。随着吸附时间的增加,萃取效率逐渐增大;当吸附时间为15 min时,吸附已经达到平衡;吸附时间进一步增大几乎对萃取效率无影响。因此,选择15 min作为吸附时间。

吸附剂在MSPE过程中提供了与目标物相互作用的作用位点,其用量会对萃取效率产生重要影响。实验选取了5、10、15、30和50 mg吸附剂,以考察其用量对萃取效率的影响。由图5c可知,吸附剂用量在5~30 mg时,敌草快的萃取效率随着吸附剂质量的增加逐渐增加;但随着吸附剂用量继续增加,萃取效率基本保持不变,故选取30 mg作为吸附剂用量。

#### 2.3.2 各种解吸参数对萃取效率的影响

在MSPE过程中,萃取效率一方面依赖于目标分析物与吸附剂之间的作用力,另一方面又取决于目标分析物与解吸剂之间的作用力,而解吸剂的种类和体积决定了分析物的解吸程度,故对萃取效率有重要影响。基于Poly([VPImi-SO_3_H][Cl])-MP在低pH条件下对敌草快的萃取效率较低,实验通过添加甲酸以提供酸性环境,选取甲醇-甲酸(9∶1, v/v)、乙腈-甲酸(9∶1, v/v)和水-甲酸(9∶1, v/v)作为解吸剂,结果如图5d所示。可以看出,相对于其他解吸剂,乙腈-甲酸(9∶1, v/v)有更高的解吸效果,故选取其为解吸剂。

实验同时考察了解吸剂体积(50~1000 μL)对萃取效率的影响,结果如图5e所示。要注意的是,解吸剂体积改变时,实验中仅依靠进10 μL样所得峰面积并不能真实反映解吸剂解吸下来的敌草快的量,难以准确判断解吸剂体积对萃取效率的影响。故图中峰面积为进样10 μL所得峰面积乘以解吸剂体积与进样体积的比值。可以看出,随着解吸剂体积从50 μL增加到600 μL,萃取效率逐渐增加,继续增加解吸剂体积,萃取效率保持不变,故选择解吸剂体积为600 μL。

用超声波振荡的方式对吸附在Poly([VPImi-SO_3_H][Cl])-MP上的敌草快进行解吸。实验选取解吸时间为20 s、40 s、1 min、2 min和3 min,考察其对萃取效率的影响。由图5f可知,敌草快可以在1 min内快速地从Poly([VPImi-SO_3_H][Cl])-MP上解吸出来,故选取解吸时间为1 min。

### 2.4 方法评价和适用性研究

在优化条件下,将Poly([VPImi-SO_3_H][Cl])-MP与MSPE和HPLC结合,对方法的性能进行了评价。敌草快在0.2~20 μg/g内具有良好的线性(*r*=0.9981),检出限(*S/N*=3)和定量限(*S/N*=10)分别为0.09 μg/g和0.2 μg/g,在低、中、高3个水平(0.4、2.0和10.0 μg/g)下,重复测定3次的相对标准偏差(RSD)为2.7%~4.2%。

用建立的方法对青菜样品进行了测定,以评价方法的适用性。样品中分别加入3个水平(0.5、1.0和2.5 μg/g)的敌草快,在优化的MSPE条件下进行测定。样品加标前后经Poly([VPImi-SO_3_H][Cl])-MP萃取后的代表性色谱图见图6。样品中未检出敌草快,加标回收率为82.7%~97.5%, RSD为2.8%~5.0%(*n*=3)。结果表明,该方法重复性好,加标回收率令人满意,是一种有效测定敌草快的方法。

**图6:**
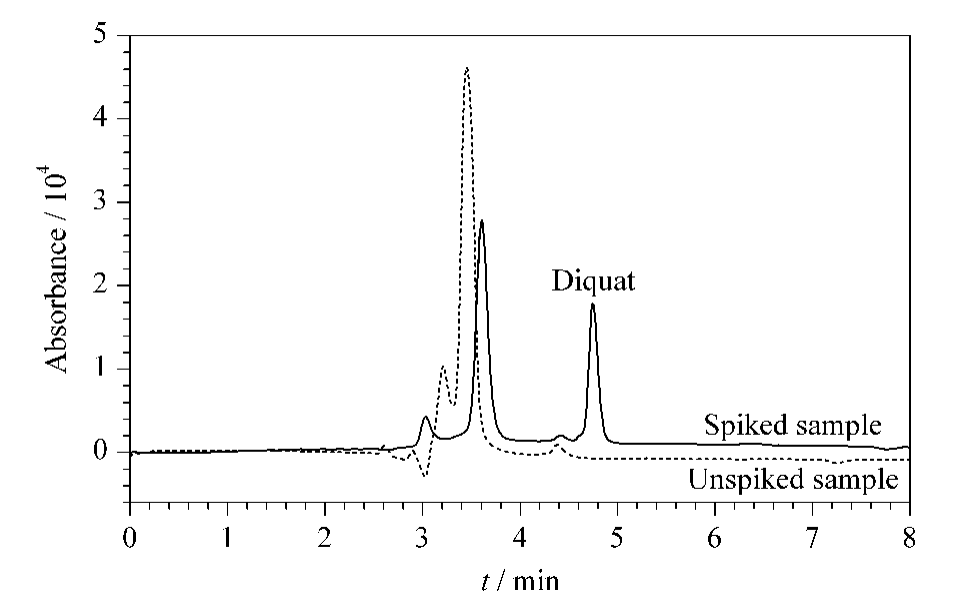
未加标样品和加标样品(2.5 μg/g)经Poly([VPImi-SO_3_H][Cl])-MP萃取后的色谱图

## 3 结论

在本研究中,采用共价聚合方式制备了磺酸功能化的聚合离子液体基磁性吸附剂Poly([VPImi-SO_3_H][Cl])-MP,采用多种手段对其结构、形貌和磁性进行了表征。磺酸基团的修饰使Poly([VPImi-SO_3_H][Cl])-MP表面在一定pH条件下带有丰富负电荷,与带正电荷的敌草快之间存在强的静电吸引作用,从而有效萃取敌草快。将Poly([VPImi-SO_3_H][Cl])-MP作为MSPE吸附剂,与HPLC技术结合,建立了一种能够准确测定青菜中敌草快的方法。研究结果为食品中极性污染物的有效萃取与测定提供了一种新途径。
